# Reflections in a time of transition: orthopaedic faculty and resident understanding of accreditation schemes and opinions on surgical skills feedback

**DOI:** 10.3402/meo.v21.30584

**Published:** 2016-04-12

**Authors:** Kenneth R. Gundle, Dayne T. Mickelson, Doug P. Hanel

**Affiliations:** 1 University Musculoskeletal Oncology Unit, Mount Sinai Hospital, Toronto, Ontario, Canada; 2 Division of Orthopaedic Surgery, Department of Surgery, University of Toronto, Ontario, Canada; 3 Department of Orthopaedics & Sports Medicine, University of Washington, Seattle, WA, USA; 4 Harborview Medical Center, University of Washington, Seattle, WA, USA

**Keywords:** competency, feedback, technical skills, Next Accreditation System, qualitative analysis

## Abstract

**Introduction:**

Orthopaedic surgery is one of the first seven specialties that began collecting Milestone data as part of the Accreditation Council for Graduate Medical Education's Next Accreditation System (NAS) rollout. This transition from process-based advancement to outcome-based education is an opportunity to assess resident and faculty understanding of changing paradigms, and opinions about technical skill evaluation.

**Methods:**

In a large academic orthopaedic surgery residency program, residents and faculty were anonymously surveyed. A total of 31/32 (97%) residents and 29/53 (55%) faculty responded to Likert scale assessments and provided open-ended responses. An internal end-of-rotation audit was conducted to assess timeliness of evaluations. A mixed-method analysis was utilized, with nonparametric statistical testing and a constant-comparative qualitative method.

**Results:**

There was greater familiarity with the six core competencies than with Milestones or the NAS (*p*<0.05). A majority of faculty and residents felt that end-of-rotation evaluations were not adequate for surgical skills feedback. Fifty-eight per cent of residents reported that end-of-rotation evaluations were rarely or never filled out in a timely fashion. An internal audit demonstrated that more than 30% of evaluations were completed over a month after rotation end. Qualitative analysis included themes of resident desire for more face-to-face feedback on technical skills after operative cases, and several barriers to more frequent feedback.

**Discussion:**

The NAS and outcome-based education have arrived. Residents and faculty need to be educated on this changing paradigm. This transition period is also a window of opportunity to address methods of evaluation and feedback. In our orthopaedic residency, trainees were significantly less satisfied than faculty with the amount of technical and surgical skills feedback being provided to trainees. The quantitative and qualitative analyses converge on one theme: a desire for frequent, explicit, timely feedback after operative cases. To overcome the time-limited clinical environment, feedback tools need to be easily integrated and efficient. Creative solutions may be needed to truly achieve outcome-based graduate medical education.

Graduate medical education in the United States is undergoing a significant restructuring of how programmes are evaluated. As part of the Accreditation Council for Graduate Medicine Education (ACGME) Outcomes Project, which previously introduced the concept of ‘six core competencies’, a shift toward more measurable trainee progression led to specialty-specific educational milestones. These ‘developmentally based, specialty-specific achievements that residents are expected to demonstrate at established intervals as they progress through training’ represent a blueprint for resident evolution toward independent practice (1).

Milestones were created by each specialty, within a rubric similar to the six core competencies. Each general competency has a number of subcompetencies, such as the orthopaedic patient care subcompetency of hip and knee arthritis (2). Increasing competency from an entry level (Level 1) to the graduation target of Level 4, and even to aspirational goals of Level 5, is defined by specific milestones that allow tracking of residents’ progression. For example, while completing an appropriate history and physical examination is a Level 1 milestone for the hip and knee arthritis patient care subcompetency, performing a primary knee and hip replacement is a Level 4 milestone.

Orthopaedics, as one of the phase I specialties, began collecting information on milestones as of July 2013. Sixteen subcompetencies related to patient care and medical knowledge were developed (Table 1), alongside assessments for interpersonal and communication skills, professionalism, practice-based learning and improvement, and systems-based knowledge (3). Each resident is to be assessed by a Clinical Competency Committee (CCC) every 6 months; the achieved milestone levels, as determined by the CCC, are then reported to the ACGME.

**Table 1:** Orthopaedic Surgery Milestones for patient care and medical knowledge

Anterior cruciate ligament
Ankle arthritis
Ankle fracture
Carpal tunnel
Degenerative spinal conditions
Diabetic foot
Diaphyseal femur and tibia fracture
Distal radius fracture
Adult elbow fracture
Hip and knee osteoarthritis
Hip fracture
Metastatic bone lesion
Meniscal tear
Paediatric septic hip
Paediatric supracondylar elbow fracture
Rotator cuff injury

The intent of the ACGME is that a variety of appropriate tools will be utilized to evaluate these diverse competencies. Carter argues that the Milestone Project was ‘intended to be the antithesis of the one-size-fits-all assessment strategy’ (4). The consensus milestone ratings by the CCC are intended to reflect the incorporation of multiple evaluations and evaluators, using methods appropriate to the subcompetency. For example, technical procedures may be evaluated in deliberate practice simulation sessions (5) or during a structured competency-based ‘boot camp’ (6). Interpersonal, professional and communication skills may also be evaluated by Objective Structured Clinical Examinations (OSCE) (7) or by 360° feedback from the healthcare team and patients. System-based practice, which includes patient safety, may be assessed by involved in quality improvement initiatives or through certificate-granting courses provided by the Institute for Healthcare Improvement's Open School (8).

One area of concern is whether milestones will be efficient and accurate, instead of burdensome, in assessing resident performance (9). Perhaps most importantly is whether the use of milestones can aid faculty in providing residents the feedback needed to improve. Validating these new tools for resident evaluation requires time, as well as input from multiple stakeholders including faculty and residents themselves.

As the reporting of Next Accreditation System (NAS) and Milestones is implemented, it is recognized that robust qualitative and quantitative research is necessary to address the impact of these programmes (9). To that end, the purpose of this study is to obtain baseline data on the knowledge of NAS and Milestones among faculty and residents in an orthopaedic residency programme, which will facilitate later studies of its acceptance and understanding. Furthermore, this transition in the paradigm of assessment is an opportunity to assess faculty and resident perspectives on current systems of evaluation. As programmes adapt, it would be ideal to implement feedback and assessment systems that also address deficiencies as identified by residents and faculty members.

## Methods

At a single, large academic orthopaedic residency programme, all residents in postgraduate years (PGY) two through five and all faculty were anonymously surveyed in November 2013 about familiarity with the NAS, Milestones, and Core Competencies as well as the topic of surgical skills feedback. This study was determined exempt by the institutional review board. The surveys were delivered via email links and were completed voluntarily and anonymously online. Answers were given on a 5-point Likert scale and included two open-ended questions for optional comments. No demographic information was recorded.

In the residency, at the time of the survey, a faculty member completes online evaluations of residents at the end of each rotation. These evaluations are passively emailed to the faculty through an automated centralized online evaluation system. Assessments are based on the categories outlined by the six core competencies. There is also the ability to enter additional comments. Faculty members and residents are encouraged to discuss feedback face to face. An internal audit assessed the time between a rotation's end and a faculty member submitting the end-of-rotation assessment form.

For statistical analysis, Likert responses were treated as ordinal data, and preplanned tests performed with nonparametric and distribution-free methods. Descriptive statistics display the median and report frequencies and percentages for answer responses. The Mann–Whitney U test was used to compare central tendencies, and Fisher's exact test to evaluate contingency tables. Statistical testing performed with Stata v11.0 (College Station, Texas).

Qualitative analysis of the optional comments was performed via a constant-comparative method by an author familiar with the technique (KG). The goal was to explore themes, as well as the range of responses provided by residents and faculty. Whenever possible, the respondents own words are used to let the data ‘speak for itself’. The authors reviewed the comments iteratively, and the final analysis agreed to by consensus.

## Results

In a programme of 40 residents (eight interns and 32 residents between postgraduate years 2 and 5), online surveys were completed by 31/32 non-intern residents (97% response rate). Interns are considered under the purview of general surgery, so were excluded from this study (10). Out of 53 faculty members, 29 completed the online surveys (55% response rate). No demographic variables were collected to ensure anonymity.

### Familiarity with evaluation systems

Faculty members and residents reported greater familiarity with the six core competencies than the NAS (Table 2). Only one resident (3%) and two faculty members (7%) reported being not at all familiar with the six core competencies. Overall, there were similarly high degrees of self-reported knowledge between the two groups (*p*=0.12). In contrast, before an organized presentation on the NAS, 10/31 (32%) residents were not at all familiar with the new system, while faculty members responded with greater familiarity (*p*=0.003). Perhaps reflecting the natural progression from the six core competencies to the NAS, after residents received a brief 10-min presentation on the NAS, there was a significant increase in self-reported knowledge of the programme (*p*=0.001) and no difference between faculty and resident familiarity (*p*=0.99). When specifically asked about the ACGME Milestones, 68% (21/31) of residents and 62% (18/29) of faculty reported at least being somewhat familiar, and there was no difference in the central tendency of responses (*p*=0.72).

**Table 2:** Familiarity with accreditation and evaluation systems

		Frequency (%)					
			
Question	Group	Not at all	Just a little	Somewhat	Very	Completely	*p*
How familiar are you with the Next Accreditation System?	Residents (pre)	10(32)	7(23)	11(34)	3(10)	0(0)	
	Residents (post)	0(0)	3(12.5)	15(62.5)	4(17)	2(8)	0.001
	Faculty	3(10)	4(14)	11(38)	9(31)	2(7)	0.003, 0.99
How familiar are you with the six core competencies?	Residents	1(3)	8(26)	12(39)	8(26)	2(6)	
	Faculty	2(7)	4(14)	6(21)	14(48)	3(10)	0.12
How familiar are you with the 16 Orthopaedic Milestones?	Residents	4(13)	6(19)	17(55)	3(10)	1(3)	
	Faculty	4(14)	7(24)	10(34)	7(24)	1(3)	0.72

### Perceptions on surgical skills feedback

**Fig. 1:**
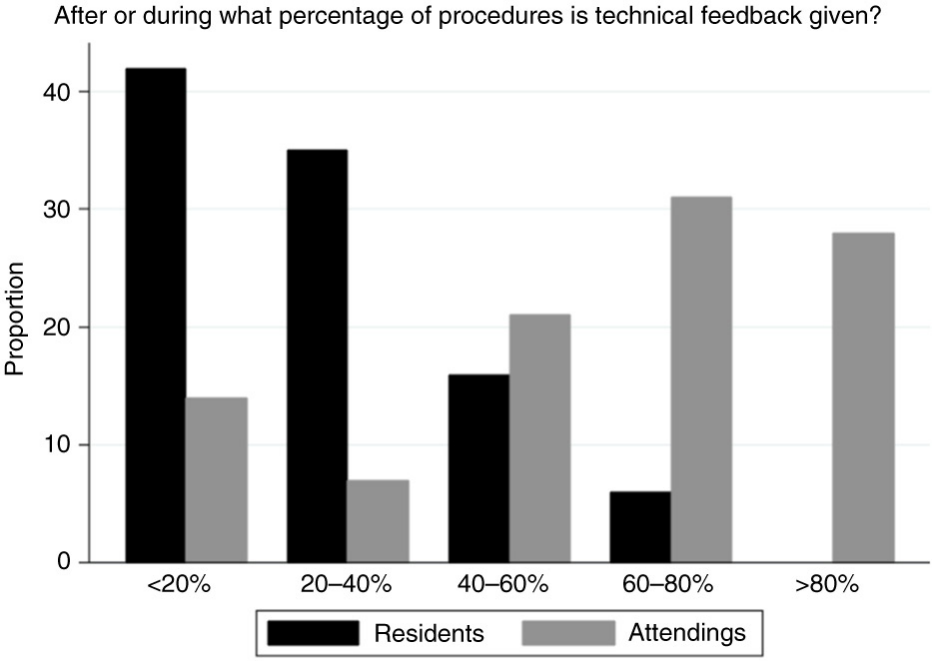
Resident and faculty self-report on the frequency of technical feedback after surgical procedures.

There were divergent results between residents and faculty regarding perceived frequency and satisfaction with surgical skills feedback (Table 3). Residents reported low frequencies of immediate feedback, with 42% (13/31) of residents reporting that surgical cases were accompanied by immediate technical skills feedback less than 20% of the time. In contrast, only 14% (4/29) of faculty reported this lowest quintile of immediate feedback. No resident reported receiving immediate technical feedback in 80–100% of cases, while 28% (8/29) of faculty reported this highest quintile. Residents reported significantly lower rates of immediate technical feedback than the faculty (*p*<0.001, Fig. 1). While no residents or faculty were completely satisfied with the quantity of feedback for surgical skills, the residents indicated less satisfaction (*p*<0.002), with 16% (5/29) being not at all satisfied. The majority of residents and faculty felt that the current, six core competency–based end-of-rotation evaluations do not provide adequate feedback and assessment of resident surgical skills (77% vs. 55%, *p*=0.1).

**Table 3:** Frequency and satisfaction with technical feedback

		Frequency (%)					
			
Question	Group	Less than 20%	20–40%	40–60%	60–80%	80–100%	*p*
After or during what percentage of procedures do you receive/provide technical feedback?	Residents	13(42)	11(35)	5(16)	2(6)	0(0)	
	Faculty	4(14)	2(7)	6(21)	9(31)	8(28)	<0.001
		Frequency (%)					
			
		Not at all	Just a little	Somewhat	Very	Completely	

How satisfied are you with the feedback on technical and surgical skills provided?	Residents	5(16)	13(42)	9(29)	4(13)	0(0)	
	Faculty	0(0)	5(17)	17(59)	7(24)	0(0)	0.002

While reporting overall agreement on the inadequacy of end-of-rotation feedback for technical skills, the groups disagreed on the promptness of receiving end-of-rotation evaluations. When asked whether online end-of-rotation evaluations are completed in a timely manner, 72% (21/29) faculty members responded ‘yes’. A majority of residents (58%, 18/31) responded that these evaluations are never or rarely completed in a timely manner (see Table 4). Regarding timeliness, further investigation supported the residents’ impression. A concurrent departmental review of 1,556 faculty evaluations over 4 years showed that less than 20% of evaluations are received within a week of the rotation ending, and more than 30% are received more than a month after the rotation ended. The average time from rotation end to receipt of written feedback was 43 days.

**Table 4:** Timeliness of evaluations

		Frequency (%)				
		
Question	Group	Never	Rarely	Sometimes	Usually	Always
Do you feel that the attending end-of-rotation feedback evaluations are completed in a timely fashion after you finish the rotation?	Residents	5(16)	13(42)	8(26)	5(16)	0(0)
		No	Yes			
Do you feel that end-of-rotation feedback evaluations are completed in a timely fashion after a resident completes a rotation?	Attendings	8(28)	21(72)			

While residents were not satisfied with the end-of-rotation evaluations and their perceived frequency of immediate feedback, they reported a global sense of appropriately progressing in technical skills. Each resident was asked whether she or he felt that their surgical skills were progressing according to PGY level, and how they imagined the faculty felt. The results were similar (*p*=0.58, see Table 5) with 88% (27/31) of residents reporting at least ‘I think so’ to the question of whether she or he is progressing in surgical skills according to level, and 84% (26/31) answering at least ‘I think so’ to whether the faculty believe she or he is progressing according to level (*p*=0.58, see Table 5). Likewise, 82% (24/29) of the faculty responded at least ‘I think so’ to whether residents are informed regarding their progression as appropriate to the level of training.

**Table 5:** Progression according to level of training

		Frequency (%)					
			
Question	Group	No	I'm not sure	I think so	I'm pretty sure	Yes	*p*
Do you feel you are progressing in surgical skills appropriate to your level of residency?	Resident	0(0)	4(13)	12(39)	8(26)	7(23)	
Do you feel that your attendings believe you are progressing in surgical skills as appropriate to level?	Resident	1(3)	4(13)	13(42)	7(23)	6(19)	0.58
Do you inform residents whether are not they are progressing surgical skills as appropriate to their level of training?	Attendings	1(3)	4(14)	10(34)	7(24)	7(24)	–

### Qualitative analysis – residents

Sixteen of 31 (52%) residents provided written comments to the open-ended prompts. Two themes emerged: a preference for in-person feedback and a sense that end-of-rotation evaluations were not timely or as useful. An interconnected third theme was an overall desire for more direct technical skills feedback.

Regarding the first point, comments ranged from ‘We need more in-person feedback’ and simply ‘Face-to-face’, to ‘Case-by-case technical skills feedback would be ideal and is rarely done’. Another wrote, ‘What works: face-to-face feedback, immediate feedback after a case’. This type of immediate feedback was described as more ‘personal’ and helpful ‘to allow us to ask questions about certain aspects of feedback’, as well as avoiding situations where a resident thinks, ‘I don't know if the attendings think I am bad and therefore don't let me do much, or the attending is just too hands-on’. No resident commented that there was too much feedback; no resident commented that feedback was overly critical. Rather, responses shared the sentiment: ‘Definitely [would] welcome more feedback – ideally directly after the procedure would be great’, and another wrote, ‘I feel I receive very little technical feedback on a case-by-case basis’.

It was recognized by a resident that ‘Instant feedback at the end of a case can often be tricky’ due to clinical responsibilities, and another commented that attendings leaving to dictate and talk to the family at the end of cases does not facilitate a ‘formal debrief’. One response summarizes this theme, balancing a desire for routine technical feedback but understanding the challenges:

I think that in busy practice and all the tasks that residents and faculty have, it is easy to forget or otherwise not take specific time to talk about what went well and what could be improved after a case. Yet this is so important! While residents can take the lead in asking for feedback, and perhaps should, ideally this would be so standard as to happen at the end of every case, and before every case.

Some of the issues residents described with end-of-rotation evaluations connected to this desire for more in-person feedback. One resident suggested, ‘I think feedback should be at more set times – every week or so’ so that changes can be made, rather than finding out at the rotations end that an attending had different expectations. Another wrote, that in contrast with case-by-case feedback, ‘The end-of-rotation feedback evaluation is suboptimal for procedural assessments’. Others describe these evaluations as ‘vague’. As mentioned repeatedly, these evaluations after the fact are even less useful when not received in a timely fashion. One resident felt that ‘When feedback is late it is useless’, and several residents doubted the veracity of procedural skills evaluation completed months after being on service with the evaluator. Instead, to facilitate resident education, ‘More timely feedback, either electronically or in-person soon after the procedures would be best and allow for real-time improvements’.

Some residents had been surprised by written evaluations that seemed disconnected from what they had been told in the course of the rotation. And there was variability suggested in the feedback patterns among the attendings: ‘It is the few that take the time to actually sit down part way through the rotation to discuss your skills that provide the best feedback’. In contrast, one resident wrote that it seemed sometimes attendings are just ‘checking boxes’ when doing the evaluations. The following comment describes both a problem with these evaluations and suggests what their ideal goal should be:

Faculty evaluations often come 3 months late or more, and if they were ever going to be helpful, it certainly isn't at that point. An end-of-rotation evaluation should ideally summarize the comments and feedback that a resident has been receiving throughout the rotation, such that it should never be a surprise – I think that for a faculty member/resident team doing evaluations well, the resident should basically be able to predict exactly what the end evaluation will be.

From the resident perspective, a summative message was, ‘a sit down face-to-face where you discuss strengths and weaknesses and how to improve is the most important’.

Residents consistently described end-of-evaluations as untimely, vague, susceptible to meaningless ‘box-checking’ and less helpful to improve technical performance. There was a desire for more feedback, ideally in-person, and case-by-case in order to facilitate improvement and provide ‘reassessment throughout the rotation’.

### Qualitative analysis – faculty

Nine of 29 (31%) faculty members responded to an open-ended question on resident evaluations. The comments were more varied in their scope and included thoughts on the timing of resident evaluations, barriers to giving appropriate feedback, and critiques of the Milestone-based system.

On the timing of evaluations, one attending seemingly contrasted with the resident sentiment, explaining that a particular group of faculty had decided to come together and reach consensus on resident performance and then meet with the residents. That attending felt, ‘We are more likely to be helpful and appropriately critical when we discuss together than do it as solitary surgeons’. However, there were also responses consistent with the residents’ theme of case-by-case feedback. Two attendings noted their own commitment to providing feedback in real time, and another wrote, ‘A culture of prompt and immediate feedback would be helpful’.

One faculty member, like the residents, noted the variability of attending evaluation styles and commented that ‘I have no idea what others are doing in the way of feedback’. As with the residents, no faculty member noted an overabundance of evaluation.

Another theme was barriers to resident evaluation, such as the length of time spent with residents. Two respondents noted their limited exposure to residents, and another wrote, ‘It is difficult to assess and improve on surgical abilities during very short rotations’ but noted balancing rotation length with subspecialty exposure and providing a similar resident experience. An additional barrier mentioned was the identification of a struggling resident early on, rather than at in the final years of the programme. A faculty member wrote, “I think that earlier evaluation of ‘endowed and taught surgical ability’ should be attempted in the R2 and R3 levels. To discover a ‘dangerous resident’ in the R4 and R5 level is terrifying and awkward!”

The most consistent theme was a variety of critiques of the Milestone-based evaluations. One commented, ‘I am able to give verbal same day feedback on the day's cases, but the written feedback in our new milestone [evaluation] is lacking’. Another wrote, ‘the milestones related to the procedures tend to focus on facts or information related to the diagnosis’. This lack of focus on technical skills was highlighted by one faculty member, who wrote out his or her own criteria for assessing residents, which included:

Does the resident display knowledge and understanding of the procedure as described in the literature?

Is economy of motion present?

Is there gentle and appropriate handling of tissues?

Is manual dexterity displayed?

Is adequate exposure and vision of important structures achieved?

Is the resident appropriately engaged in the procedure?

Is the resident able to assess his or her progress and make modifications as the situation might require?

A further critique of the orthopaedic subcompetencies was that they only exist for a limited number of procedures, such that many faculty members might never perform an applicable case with a resident yet still have valuable insight and feedback on technical skills.

## Discussion

Our study combined quantitative and qualitative methodologies to evaluate resident and faculty understanding of the new Milestone-based NAS, as well as perspectives on current systems for evaluating surgical skills in a single, large orthopaedic surgery programme. As programmes shift evaluation systems to meet accreditation requirements, our results suggest that a focus on frequent, explicit, immediate feedback that fits into the fast-paced clinical environment would best address the deficiencies noted by residents.

Reflecting its years of implemented use in resident evaluation, both the faculty and residents were similarly familiar with the six core competencies (*p*=0.12). While residents were initially less familiar with the NAS and its milestones (*p*=0.003), after a brief presentation resident understanding increased (*p*=0.001) to be equivalent to the faculty (*p*=0.99). This data shows evolving knowledge of the orthopaedic milestones and NAS, as well as established familiarity with the six core competencies. This baseline data will allow tracking of resident and faculty knowledge of these new accreditation paradigms.

The quantitative and qualitative analyses converge on one theme: residents desire frequent, immediate, specific feedback. Residents were significantly less satisfied than faculty with the amount of perceived technical and surgical skills feedback being provided (*p*=0.002). While 28% of faculty reported that technical feedback was given to residents in 80–100% of cases, none of the residents agreed; residents reported receiving feedback in significantly fewer cases than what the faculty reported (*p*<0.001, Fig. 1). This may reflect faculty and resident differences in what constitutes specific feedback (11), and these findings are in lines with multiple other specialties (12, 13). For example, a survey of general surgery residents likewise found a perception gap between trainees and faculty members, with a desire by residents for more immediate feedback (10). One explanation is that informal and unstructured comments may be insufficient, and that feedback should be explicit. A potential solution is to utilize written tools, such as surgical skills feedback (SurF) cards, which have shown increased resident satisfaction (14).

While additional explicit written assessments may address the feedback perception gap, any solution must account for the hectic clinical environment. Barriers to additional face-to-face feedback, according to the residents in this study, include busy clinical practice and the need to efficiently complete tasks between operative cases. The faculty also mentioned barriers of relatively short clinical rotations, limited exposure to residents and the need to identify struggling residents early in training. Similar challenges to resident feedback have been noted in other fields (10). A key element of a feedback tool is its promptness and ease of integration into practice. Given the current pace of clinical practice, programmes adapting to the NAS should recognize the need for real time, rapidly completed evaluations (14).

Residents and faculty agreed that end-of-rotation evaluations are insufficient, especially for technical skills. Overall, 77% of residents and 55% of faculty felt that end-of-rotation evaluations are not adequate for the assessment of resident surgical skills (*p*=0.1). Though the Milestones were designed for programme accreditation purposes, some programmes are integrating milestone-based tools into end-of-rotation resident evaluations (15). Programmes utilizing milestones in this way are reporting less ‘grade inflation’ and greater inter-resident discrimination compared to core competency–based end-of-rotation evaluations (15). This practice has been described as an ‘unexpected consequence’ of the NAS, which aims to have multiple evaluation tools culminate in consensus milestone level determinations biannually by the CCC (4). This may reflect programmes attempting to comply with the NAS requirements in the time and resource constrained clinical environment. Our results suggest that simply changing the format of end-of-rotation evaluations will be insufficient to provide residents the feedback they most desire.

Residents also reported frequent delays in receiving these evaluations, in contrast with attendings. Review of department records corroborated that less than 20% of evaluations are received within a week of completing a rotation, and more than 30% are received over a month after the rotation ends. This delay in written evaluation limits their usefulness for trainees and was a source of dissatisfaction among the residents.

Although the goal of competence-based education is multifaceted, residents also value knowing simply whether or not they are on ‘the right track’. For example, a junior resident achieving a Milestone Level 2 may or may not represent the appropriate progress for their level of training. Only 4/31 (13%) residents were sure if their surgical skills were appropriate to level, and they did not perceive a difference in what the attendings’ thought (*p*=0.58). Consideration should be given to providing residents a more generalized global rating of below, at or above appropriate progress at regular intervals. This may also address faculty concerns about identifying and intervening early with residents having difficulty (16).

This study has several limitations. While 31/32 residents between PGY 2–5 participated (97% response rate), only 29/53 faculty members returned surveys (55% response rate). It is possible that the faculty members who elected to complete the surveys differ from those who did not. This may have impacted the quantitative analysis, as well as the breadth of responses on the qualitative analysis. Also, the results found in this one programme cannot be assumed to be representative of other orthopaedic residencies nor other specialties.

As programmes utilize the window of opportunity provided by the rollout of the NAS to institute broader residency evaluation reforms, the results of this study of orthopaedic surgery residents and faculty provide guidance. If graduate medical education is to make the desired transition from process focus to an outcome-based focus (17), input from frontline faculty and residents is necessary. Simply repurposing end-of-rotation evaluations to incorporate the milestones may not increase resident feedback satisfaction, have the desired impact on tracking performance or provide optimal data for review. It also fails to embrace the multifaceted assessments the ACGME sought to encourage with the NAS. Residents consistently desired more immediate, explicit, timely feedback after procedures. Given the barriers in clinical practice that limit organic face-to-face instruction, feedback tools will also require creativity to successfully be integrated in a busy environment without becoming onerous.
